# From Ancient Philosophy to Endosymbiotic Theory: The Bacterial Origin and Key Role of Mitochondria in Immune Responses

**DOI:** 10.3390/microorganisms13092149

**Published:** 2025-09-15

**Authors:** Alexandra Mpakosi, Christiana Kaliouli-Antonopoulou, Vasileios Cholevas, Stamatios Cholevas, Ioannis Tzouvelekis, Maria Mironidou-Tzouveleki, Emmanuel A. Tsantes, Deny Tsakri, Marianna Vlachaki, Stella Baliou, Petros Ioannou, Rozeta Sokou, Stefanos Bonovas, Andreas G. Tsantes

**Affiliations:** 1 Department of Microbiology, General Hospital of Nikaia “Agios Panteleimon”, 18454 Piraeus, Greece; 2 Department of Immunology, General Hospital of Nikaia “Agios Panteleimon”, 18454 Piraeus, Greece; ckalanto@gmail.com; 3 School of Medicine, University of Bologna, 40126 Bologna, Italy; billcholevas34@gmail.com; 4 School of Pharmacy, European University of Cyprus, Diogenes 2404 Engomi, P.O. Box 22006, Nicosia 1516, Cyprus; stam17112004@gmail.com; 5 School of Agricultural Technology, Food Technology and Nutrition, Alexander Technological Educational Institute of Thessaloniki, 57400 Thessaloniki, Greece; tzouvelekisgiannis@yahoo.gr; 6 Department of Pharmacology, School of Medicine, Faculty of Health Sciences, Aristotle University of Thessaloniki, 54124 Thessaloniki, Greece; mmyronidauth@gmail.com; 7 Laboratory of Haematology and Blood Bank Unit, School of Medicine, “Attiko” Hospital, National and Kapodistrian University of Athens, 12462 Athens, Greece; manolistsantes@yahoo.com; 8 Department of Microbiology, Medical School, National and Kapodistrian University of Athens, 11527 Athens, Greece; denytsakris@gmail.com (D.T.); mariannavl03@gmail.com (M.V.); 9 Laboratory of Toxicology, School of Medicine, University of Crete, 71003 Heraklion, Greece; stellabaliou@gmail.com; 10 School of Medicine, University of Crete, 71003 Heraklion, Greece; p.ioannou@uoc.gr; 11 Neonatal Department, Aretaieio Hospital, National and Kapodistrian University of Athens, 11528 Athens, Greece; sokourozeta@yahoo.gr; 12 Department of Biomedical Sciences, Humanitas University, Pieve Emanuele, 20072 Milan, Italy; stefanos.bonovas@hunimed.eu; 13 IRCCS Humanitas Research Hospital, Rozzano, 20089 Milan, Italy; 14 Microbiology Department, “Saint Savvas” Oncology Hospital, 11522 Athens, Greece

**Keywords:** endosymbiosis, mitochondrial DNA, immune responses, proinflammatory signaling pathways, inflammasome, cellular homeostasis, evolution, eukaryotic cell

## Abstract

The endosymbiotic theory, which is the crucial starting point of eukaryogenesis, was first mentioned in the philosophy of the pre-Socratic Greek philosopher Empedocles. According to him, everything merges into units with differential survival. Similarly, during eukaryogenesis, the fusion of two distinct units resulted in the creation of a new cell type that possessed a newly formed organelle, the mitochondrion. Since then, the mitochondrion has been a key regulator of health and immunity. Furthermore, many of its characteristics and functions are due to its endosymbiotic bacterial origin. For example, it possesses damage-associated molecular patterns that can activate inflammatory signaling pathways, has circular DNA with CpG-rich motifs, as well as a double phospholipid membrane, and divides by fission. Mitochondrial function plays a critical role in maintaining cellular homeostasis, as they meet the cell’s energy needs and regulate many of its functions. However, after cellular damage due to infection, radiation, or toxins, mitochondrial stress and dysfunction can occur and mitochondrial DNA can be released into the cytosol. Cytosolic mitochondrial DNA can then activate proinflammatory signaling pathways, mediated by TLR9 and cGAS, as well as inflammasomes, triggering inflammation and autoimmunity.

## 1. Introduction

The ancient Greeks were particularly concerned with the origin of the world. In the Theogony, the epic poem by Hesiod (7th century BC), it is stated that in the beginning Chaos appeared, who gave birth to Earth (represented matter), Tartarus (represented time and death) and Eros (creation). In particular, the pre-Socratic Greek philosophers, although not as famous, attempted to decipher important questions about the origin of life to provide answers to how order emerged from chaos, but also, most importantly, how this order and the stability of life were maintained within an ever-evolving context. They wondered how it was possible for species to reappear with the same or even greater organization, diversity, and complexity while they were in a state of constant evolution and change [1]. This is exactly what Heraclitus (544–483 BC) argued when he said “*ta panta rei*” (“everything flows”), meaning that everything changes and everything remains the same through mutual influence. Pythagoras (581–497 BC) also argued that nothing is absolutely new and that everything changes cyclically, meaning that everything that exists can be reborn within a specific cycle [2]. On the other hand, according to Parmenides’ theory (515–470 BC), regardless of the change of external things, “*to einai*”, meaning every being, is the only object of Truth; Parmenidean Truth on the one hand accepts the movement and diversity of the world but, on the other, emphasizes its unity and continuity [2].

Nevertheless, Empedocles of Acragas (495–435 BC) stands out as one of the most important figures of pre-Socratic philosophy, a true pioneer of ancient Greek thought. His philosophical treatise was discovered when, in 1904, German archaeologist Otto Rubensohn purchased a roll containing 52 papyrus fragments from antiquities shop in Akhmim, Egypt. The roll had originally been found with a mummy discovered in the necropolis of the ancient city of Panopolis. The text of the roll was written in columns measuring 30 hexameters each. It was not transcribed or translated until 1992, when the papyrologist Alain Martin attributed it to Empedocles. Until then, the papyrus had remained in the National Library of the University of Strasbourg [3].

Interestingly, according to one view, Empedocles is actually considered the first scientist in the West [2]. However, he belongs to an era in which there was still no real distinction between the magician, the poet, the soothsayer, the shaman, the physician, and the philosopher. He seems to have written two studies in verse, *Peri physeos* (On Nature), which deals mainly with natural philosophy, and *Katharmoi* (The Purifications), which has a religious, mystical, and occult character. His work is enriched by his intense imagination and is characterized by his multifaceted personality. In particular, in the field of medicine, it seems that he had realized the role that the patient’s mental state plays in diseases. In fact, it is rumored that he used music as a means of mental upliftment, to heal pain and sadness. Similarly, during a plague epidemic that broke out in the city of Selinunte, Empedocles had realized very early that the cause of the infection was the stagnant waters of the area. Thus, he saved the city by organizing a plan to divert the flow of the two adjacent rivers, which were responsible for the stagnant waters [4]. His complex personality, his intelligence, his life dominated by mysterious stories and miracles, and his bold philosophical views quickly led him to the realm of legend. Indeed, Nietzsche described him as the most diverse figure of archaic philosophy and recognized that in him two worlds collide, that of myth and that of science. In the philosophy of both Empedocles and Nietzsche, nature is given priority. Both believe in the continuous birth and death through the blending and acceptance of existence and the union between self, others, and nature [5].

It seems that the endosymbiotic theory, which is now accepted as the crucial starting point of eukaryogenesis, was first mentioned in the philosophy of Empedocles. In fact, he was the first to support the idea of parts aggregating into units with differential survival. Indeed, it is now widely accepted that during eukaryogenesis the fusion of two distinct units resulted in the creation of a new cell type. Furthermore, the newly formed organelle of this new cell, the mitochondrion, appears to be a key regulator of human health and immunity. Interestingly, many of the characteristics and functions of the mitochondrion are due to its endosymbiotic origin. Therefore, with the present review we will attempt to develop all of the above, connecting the Pre-Socratic philosophy with the mitochondrial endosymbiosis, and we will try to analyze how the latter critically regulates health and immune responses.

## 2. Empedocles’ Theory of Evolution

**Figure 1:**
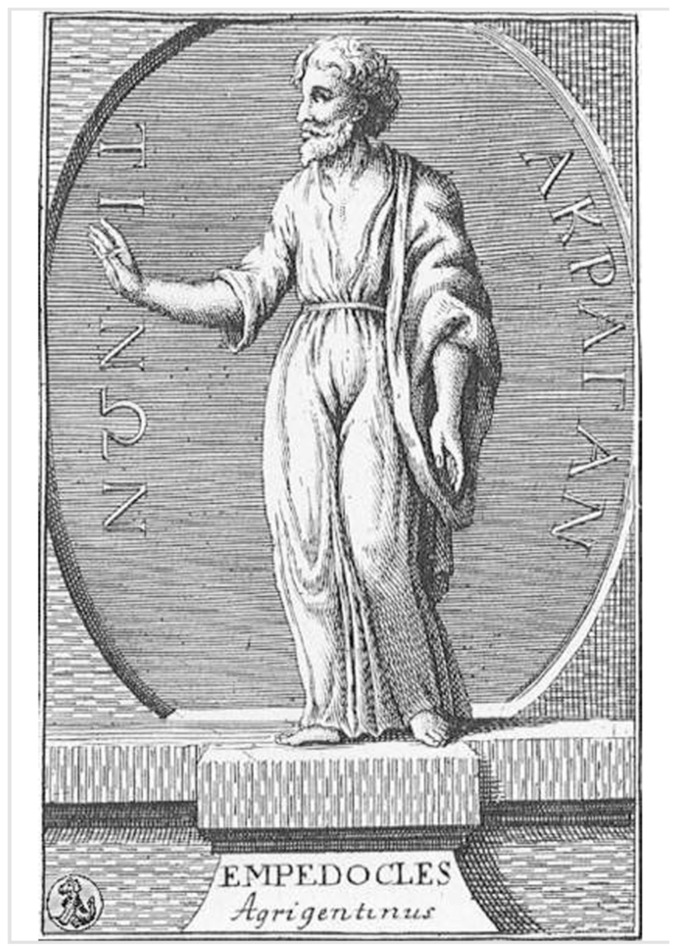
Empedocles. Source: http://www.phil-fak.uni-duesseldorf.de/philo/galerie/antike/empedok.html, accessed on 8 August 2025 (public domain).

Empedocles argued that there were four elements (air, water, earth, and fire), which he called “*rhizomata*” (roots), as the primary substances that existed from the beginning of the world. These elements could cause change by interacting with each other, but at the same time remain the same indestructible and unchanging (Figure 1).

According to Empedocles’ theory, constant mixing and separation produced everything. He gave priority to the principles of nature that caused constant birth and death through mixing and evolution. The entire philosophy of Empedocles is based on these basic principles. From these four elements and under the influence of two forces acting on them, *Philotis* (attractive force) and *Neikos* (repulsion), human tissues were formed and the first humans were created. The entire world is alternately created and destroyed in an eternal cyclical process. Within it, everything is united and in harmony under the influence of *Philotis*, while gradually this harmonious bond is broken under *Neikos* until the latter dominates completely, resulting in absolute multiplicity. Then, harmony gradually returns under the power of *Philotis* and the same pattern is repeated, representing the creation and destruction of a world [6]. In other words, on the one hand the unification of beings occurs through the *Philotis*, and, on the other hand, their evolution and differentiation occur under the influence of *Neikos*. According to modern views, the above is reminiscent of the basic theories of evolutionary biology also recorded by Charles Darwin in his book “The Origin of Species”. According to him, all species are descended from common ancestral species and evolve through modifications by a process he called natural selection which acts on the differences that exist in individuals of the same species [7,8,9]. Beings reproduce and “double” as they grow [7,8]. Natural selection comes to counteract growth either through cessation of reproduction or through death. However, Charles Darwin himself had rejected Empedocles’ views to support the correctness of his own scientific theory in the Victorian era, during which hypotheses about evolution were dominated by the Christian theory of the origin of the world [10].

Similarly, Aristotle had downplayed the philosophical views of Empedocles. In fact, unlike him, Aristotle believed that the soul is the cause and purpose of existence:

“And Empedocles has not spoken correctly, when he added this, that growth occurs in plants, downwards because they have roots and because the earth behaves in this way by its nature, and upwards because of fire in the same way. Moreover, what is it that holds fire and earth together, which move in opposite directions? For this (the plant) will disintegrate unless there is something to hinder it; and if there is, that is the soul, the cause of both growth and nourishment”. Perι psychιs (On the soul) by Aristotle.

With this observation, Aristotle judged Empedocles’ view that growth is due to rhizomes as incorrect, because in this case the growth of everything would be without limits. For Aristotle, these limits are set by the soul as the cause of the movement, evolution, and form of beings [11].

Furthermore, Aristotle rejected Empedocles for his surreal descriptions, arguing that his study was not governed by seriousness [10]. However, more and more researchers agree that Empedocles’ work constitutes a description of biological evolution about 2400 years before Darwin. Indeed, growth and differentiation are key features of modern evolutionary and systems biology. For example, Caetano-Anollés G. and Janko R. have argued that his philosophy represents a biphasic model of unit creation in biological systems characterized by *accretion* and simultaneous change [7,12]. According to this theory, the loose and disorganized connection of the parts of the first phase is followed by their differentiation, and their interaction with relations of competition and selection, resulting in the transformation of the parts into units with close connection. In the second phase, the differentiation of the module variants and their integration into a new higher-level organizational cycle follow [12]. Thus, according to the authors, Empedocles’ philosophy implies concepts such as natural history and systematization, natural selection, *accretion*, units, life cycles, and evolving networks [7].

## 3. From Empedocles to Endosymbiosis

Empedocles’ philosophical views on the origin of life were revived much later, in 1967, when Lynn Margulis published an article entitled “On the Origin of Mitosing Cells” in the Journal of Theoretical Biology [13]. Influenced by the pre-Socratic philosopher’s views that the first beings on Earth had merged and from these others had reproduced through natural selection, Lynn Margulis further demonstrated that the endosymbiotic processes had played a critical role in the origin and evolution of eukaryotic cells [14]. In fact, the term endosymbiosis in evolutionary biology describes the evolutionary process through which a symbiotic relationship, that is, the close and long-term interaction between different biological organisms, leads to the creation of an entirely new biological structure. Several other scientists had also formulated such theories, including the German Anton de Bary, who dealt with symbiosis in lichens, the French Paul Portier and Ivan Wallin, the Russians Andrei Famintsyn and Konstantin Mereschkowsky, who used this term for the first time, and Boris Kozo-Polyansky, whose archives were confiscated after his death in 1957 by the KGB [15].

**Figure 2:**
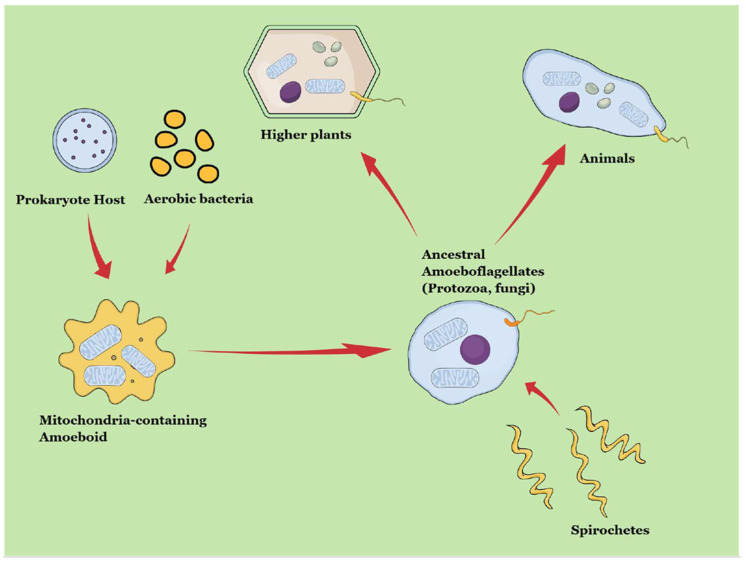
Lynn Margulis’s symbiogenetic theory.

Margulis, however, mainly supported the endosymbiotic bacterial origin of mitochondria and plastids as well as the origin of the eukaryotic flagellum and mitotic apparatus from a possible endosymbiotic spirochete-like organism (Figure 2) [13,14]. The key concepts regarding origin of life linking Empedocles’ philosophy to the endosymbiotic theory throughout history are summarized in Table 1.

**Figure 3:**
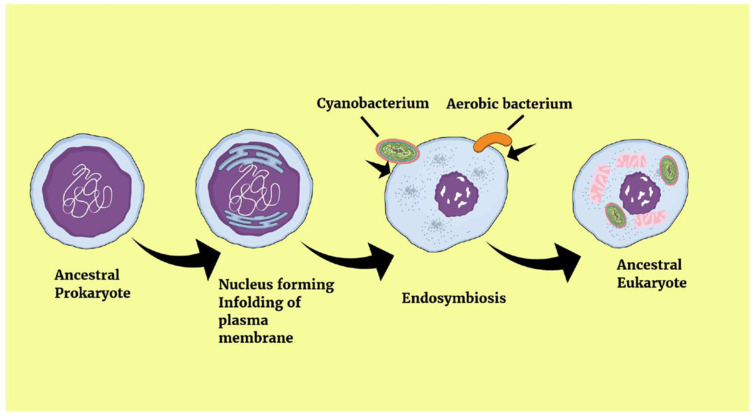
The endosymbiotic theory.

Modern researchers have now accepted the endosymbiotic theory of the origin of organelles, mainly mitochondria and plastids. It is argued, in fact, that the mitochondrion and the plastid have a separate, endosymbiotic origin. The plastid, in particular, appears to have originated from an endosymbiotic cyanobacterium and to have occurred in an ancestor of the Archaeoplastids, the eukaryotic lineage that contains land plants and algae. Subsequently, it seems that plastids entered into other species of algae, and then plants, through a process of secondary symbiosis (i.e., eukaryotic hosts recruited eukaryotic symbionts) [14,16]. It appears that mitochondria were essential for this process, both to harness solar energy and to protect the chloroplasts during the night and from cellular stress. In return, the endosymbiotic chloroplast provided sugars and oxygen to the mitochondria (Figure 3) [17,18].

As has been argued, the early endosymbiosis of mitochondria and chloroplasts led to upgrades in cellular energetics and multicellularity. All later endosymbioses, such as those of plants with their microorganisms or endosymbionts in invertebrates, have been built on these first ones and have contributed to biodiversity and the complexity of life. Endosymbiotics provide new phenotypes to their hosts, conferring new evolutionary and adaptive mechanisms. Hosts, on the other hand, develop adaptive mechanisms for resource securing and cellular regulation, as well as support mechanisms for genetically degraded symbionts [19].

## 4. Endosymbiotic Origin of Mitochondria

It has been suggested that the mitochondrion is evolutionarily older than the plastid. However, it is not yet clear at what stage of evolution exactly the initial endosymbiotic process took place, how long it lasted, and how it was completed, i.e., what were the exact mechanisms that ultimately led to the creation of a fully integrated organelle. Gray MW has attempted to hypothesize this process of endosymbiosis by comparing modern organelles with their closest bacterial relatives. It would likely have been a long-term process that took place in many stages. The bacterial ancestor would first have to lose its cell wall, while the symbiotic cell would have to acquire metabolite transporters and lose genes to reduce its genome. These genes could presumably be transported to the nucleus where they could be activated and redirect their cytoplasmically synthesized protein products back to the developing organelle [14].

Two possible theories have been proposed depending on the moment of creation of mitochondria within evolutionary time from FECA (first eukaryotic common ancestor) to LECA (last eukaryotic common ancestor) [20]. According to the early model theory, the host was an anaerobic archaeon that needed hydrogen, which it found by adopting an α-proteobacterium that produced molecular hydrogen through anaerobic heterotrophic metabolism [21,22]. In this hypothesis, the origin of the mitochondrion and the origin of the eukaryotic cell occurred simultaneously [23]. According to the late model theory, on the other hand, symbiosis occurred through phagotrophy, that is, the endocytosis of the symbiotic organism by the host into a phagosome [24].

In addition, LECA appears to have possessed a fully functional mitochondrion. Furthermore, it likely also possessed a nuclear envelope, intracellular compartments, a cytoskeleton, and complex metabolic and gene regulatory mechanisms. Thus, the entire process probably could not have happened very close to the emergence of LECA, as it required a lot of time and complex procedures [25]. Then, according to one view, the acquisition of the mitochondrion seems to have led to high energy production and perhaps this contributed to the development of advanced membrane and cytoskeletal structures [26,27].

## 5. Mitochondrial Endosymbiosis

The integration of transport proteins, metabolic pathways, and other fission–fusion mechanisms with the host cell, as well as the new cristae structure, gave the mitochondria a functional and structural evolutionary advantage compared to prokaryotic cells [28]. However, due to their endosymbiotic origin, mitochondria still retain bacterial characteristics. For example, they possess many damage-associated molecular patterns (DAMPs) that can trigger inflammatory signaling pathways, which have similarities to pathogen-associated molecular patterns (PAMPs) found in bacteria. Furthermore, mitochondria, like bacteria, have circular DNA with CpG-rich motifs. In addition, they have a double phospholipid membrane, the inner one rich in the phospholipid cardiolipin and containing the mitochondrial matrix, and the outer one, just like Gram-negative bacteria [28,29]. Moreover, mitochondria divide by fission, like their bacterial progenitors. However, mitochondrial replication is not autonomous, but depends on the nucleus, where most of the necessary proteins are encoded and then imported into the mitochondrion [30,31].

Despite the above, the host cell does not perceive mitochondria as foreign stimuli capable of triggering an immune response, as they are well organized into intracellular compartments. This symbiotic interaction between mitochondria or between mitochondria and other organelles within the host cell plays an important role in maintaining cellular homeostasis [32].

During their evolution over time, mitochondria have transferred many of their genetic functions to the nucleus of the host cell. However, as already mentioned before, they maintain their own genome, which can be degraded, and mitochondrial activity can be dramatically affected if the endosymbiosis is disrupted. There are several mechanisms that potentially cause disruption of mitochondrial symbiosis. Oxidative stress, which leads to increased production of ROS (reactive oxygen species) with subsequent damage, loss of mitochondrial membrane function, and reduced ATP synthesis, is one of them. Additionally, mitochondrial mutations can lead to dysfunction of the electron transport chain, further disrupting intracellular metabolic processes [32,33]. It has been suggested that such mutations in mitochondrial DNA may contribute to the pathogenesis of cancer, diabetes, cardiovascular disease, pulmonary hypertension, aging, and neurodegenerative diseases such as multiple system atrophy, Alzheimer’s, and Parkinson’s diseases [34,35,36,37,38,39,40,41]. What actually happens is that in such cases where endosymbiosis is disrupted, the mitochondrial dysfunctions that occur dramatically affect the cell and its processes, including its metabolism, proliferation, apoptosis, and quality control [37]. Furthermore, mitochondrial DNA variants may be associated through epigenetic mechanisms with differences in methylation levels and gene transcription in the nuclear genome [42].

Moreover, interactions between nuclear and mitochondrial genomes also appear to influence disease phenotypes. Thus, a disruption of endosymbiosis likely disrupts coordinated gene expression [43]. In addition, given that nuclear DNA is what encodes the majority of mitochondrial proteins and regulates mitochondrial function, it follows that mutations or epigenetic changes in the nuclear genome can then affect the mitochondrial genome, leading to instability or even depletion, subsequently causing mitochondrial disease [37,44,45,46].

## 6. Mitochondria as a Key Regulator of Immune Responses

### 6.1. Mitochondria and Inflammation

Although mitochondria retain their own DNA, it differs from nuclear DNA. Thus, mitochondrial DNA, due to its bacterial origin, resembles the DNA of prokaryotic cells. It is a circular, double-stranded DNA molecule formed by the heavy and light chains, without histones, and organized into tight nucleoprotein structures, the nucleoids [47]. One of the proteins that make up nucleoids, mitochondrial transcription factor A (mtTFA or TFAM), appears to be immunostimulatory [48]. Furthermore, mitochondrial DNA appears to be hypomethylated or aberrantly methylated compared to nuclear DNA [49,50,51]. In particular, due to its origin, mitochondrial DNA likely harbors unmethylated CpG patterns similar to bacterial DNA, which could potentially have the ability to activate pattern recognition receptors such as TLR9, triggering a proinflammatory signaling pathway dependent on NF- κB (Nuclear factor-κB) [52,53,54].

Indeed, cellular damage, due to infection, radiation, or toxins, can induce mitochondrial stress and the release of mitochondrial DNA into the cytosol [55]. Then, cytosolic mtDNA can activate pathways mediated by TLR9 (Toll-like receptor 9) and cGAS (cyclic GMP-AMP synthase), as well as inflammasomes [55].

The development of pores in the outer mitochondrial membrane (MOMP) with the involvement of BAX and BAK proteins of the Bcl-2 family appears to contribute to this release of mitochondrial DNA [56]. Mechanisms such as the formation of the mitochondrial permeability transition pore (mPTP), the voltage-dependent anion channel (VDAC), and mitochondrial-derived vesicles (MDVs) may also be involved [55,57,58,59,60]. Furthermore, sometimes the extrusion of mitochondrial DNA into the cytosol can be the result of mutation or deletion of genes involved in the maintenance of mitochondrial structure, stabilization of mitochondrial DNA, or mitophagy [61,62].

**Figure 4:**
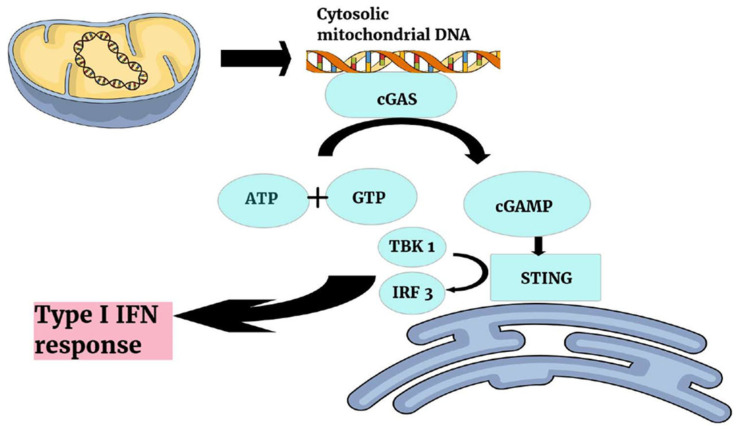
CGAS-cGAMP-STING pathway. Abbreviations: IRF 3, Interferon Regulatory Factor 3; IFN, Interferon; TBK1, TANK-binding kinase 1; STING, stimulator of interferon genes; 2′3′-cGAMP, 2′3′-cyclic GMP-AMP; cGAS, cyclic GMP-AMP synthase; AMP, adenosine monophosphate; GMP, guanosine monophosphate.

Both cyclic GMP-AMP synthase (cGAS) and 2′3′-cyclic GMP-AMP (cGAMP) are cellular mechanisms for recognizing foreign or pathogenic DNA [63,64]. Thus, once mitochondrial DNA is released into the cytosol, it is firstly detected and bound by the cGAS protein, which promotes the conversion of ATP and GTP to cGAMP. cGAMP binds in turn to the stimulator of interferon genes (STING) protein, located in the endoplasmic reticulum, activating the kinase TBK1 (TANK-binding kinase 1), which then phosphorylates the transcription factor IRF3 (Interferon Regulatory Factor 3), triggering a type I interferon response (Figure 4).

**Figure 5:**
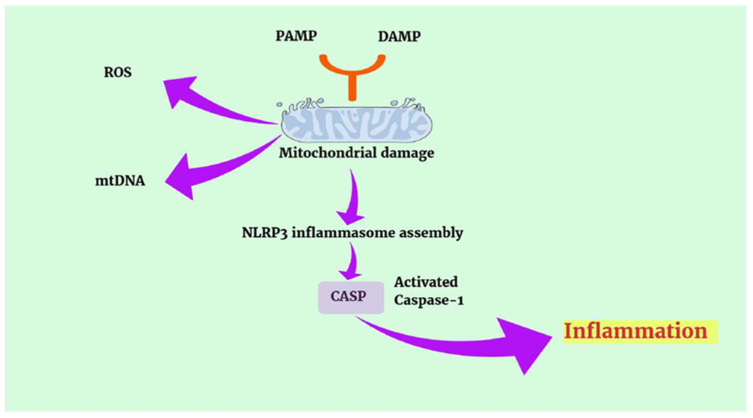
NLRP3 inflammasome activation. Abbreviations: DAMP, damage-associated molecular patterns; PAMP, pathogen-associated molecular patterns; NLRP3, NOD-like receptor family pyrin domain containing 3; CASP-1, caspase-1; ROS, reactive oxygen species; mtDNA, mitochondrial DNA.

Inflammasomes, on the other hand, are complexes composed of a PRR (pattern recognition receptor), the adaptor protein ASC (apoptosis-associated speck-like protein containing a caspase recruitment domain), and the cysteine protease caspase-1 [65]. This receptor is potentially activated by both exogenous PAMPs and endogenous DAMPs, such as in cellular stress. It may belong to NOD (nucleotide oligomerization domain) receptors, LRR (leucine-rich repeat) receptors, NLRP1 (NOD-like receptor family, pyrin domain containing 1), NLRP3, and CARD (caspase recruitment domain)-containing protein 4 of the NLR (NOD-like receptor) family (NLRC4), and is absent in melanoma 2 (AIM2) [55,66]. Mitochondrial DNA can activate the NLRP3 and NLRC4 inflammasomes, leading to caspase-1-mediated cytokine secretion and triggering inflammation (Figure 5) [55,67,68].

### 6.2. Mitochondria and Autoimmunity

Mitochondria play an essential role in ATP production through oxidative phosphorylation (OXPHOS) and are key regulators of the activation, proliferation, and function of immune cells, including monocytes/macrophages, T and B cells, and dendritic cells [69,70,71,72]. Therefore, it is expected that these processes of cell activation, proliferation, differentiation, and function are influenced by the production of mitochondrial reactive oxygen species (mtROS), the release of cytochrome c and mitochondrial DNA, and the production of metabolites [73]. ROS production, in particular, has recently emerged as an important mitochondrial function, as important as ATP production, contributing to cellular adaptation and resilience [74]. Mitochondrial symbiotic function is therefore important for cellular homeostasis, as its dysfunction increases intracellular oxidation and stress, affects the functions of other organelles such as the endoplasmic reticulum and lysosomes, and can induce autophagy, damage, and cell death [75]. In addition, they play a key role in the normal function of regulatory T cells (Tregs). Tregs are responsible for maintaining immune homeostasis and self-tolerance. To meet their energy needs, they depend more on mitochondrial oxidative phosphorylation rather than glycolysis, as is the case with conventionally activated T cells. The essential role of the mitochondrial unfolded protein response (mitoUPR) in supporting the functional integrity of Treg cells has also been highlighted [76]. Furthermore, Treg cells possibly require complex III of the mitochondrial respiratory chain for both immune regulatory gene expression and suppressive function [77].

Therefore, Tregs are particularly sensitive to mitochondrial dysfunction and imbalances. For example, it has been shown that Treg cells from individuals with autoimmunity exhibit increased mitochondrial oxidative stress and a robust DNA damage response (DDR) associated with cell death [78].

Abnormal mitochondrial metabolism has also been observed in immune cells of individuals with autoimmune diseases, including rheumatoid arthritis and systemic lupus erythematosus [79,80,81]. In such cases, mitochondrial damage and homeostatic disruption can lead, as already mentioned above, to mitochondrial DNA extrusion and increased mitochondrial ROS production, resulting in the activation of inflammatory pathways [80]. On the other hand, chronic low IFN-γ has been associated with lupus nephritis, possibly because it disrupts mitochondrial complex I activity in macrophages [82,83].

Furthermore, as has been shown in rheumatoid arthritis, molecular defects lead to inadequate DNA repair in hematopoietic stem cells, neutrophils, and naive and memory CD4+ T cells, as well as to accumulation of damaged DNA in the nucleus, telomere ends, and mitochondria. These are also followed by cell cycle abnormalities, premature loss of telomeres, and release of mitochondrial DNA into the cytoplasm, which activates the inflammasome [84]. On the other hand, rheumatoid arthritis, autoimmune hypothyroidism, and systemic lupus erythematosus have been associated with a decrease of mtDNA copy number (mitochondrial DNA copy number), which corresponds to the ratio of mitochondrial to nuclear DNA copy number and which may reflect mitochondrial DNA damage [85,86,87,88]. Additionally, it has been hypothesized that dysfunction of genes related to mitochondrial homeostasis could be involved in this reduction in mitochondrial DNA copy number [89]. On the contrary, mtDNA copy number has been associated with a reduced risk of Sjögren’s syndrome, multiple sclerosis, and systemic sclerosis. However, more studies are needed for more reliable conclusions, in which it should always be taken into account that mtDNA copy number is affected by the stage of the disease, as it fluctuates during its progression [90,91,92].

## 7. Conclusions

We could assume that when Empedocles argued that everything is united and in harmony under the influence of *Philotis*, while this harmonious bond is broken and destroyed under *Neikos*, he essentially laid the foundations of the endosymbiotic theory. The most important example of endosymbiosis during evolution is the mitochondria. Since then, mitochondrial endosymbiosis has played a key role in maintaining cellular homeostasis. Indeed, mitochondria are the cell’s energy-producing sources, and they influence many cellular functions. On the other hand, mitochondrial dysfunction can disrupt cellular function and activate inflammatory signaling pathways, triggering diseases. A thorough understanding of mitochondrial function and the mechanisms of mitochondrial involvement in disease is particularly important as targeting mitochondrial dysfunction has emerged as a promising therapeutic approach and has shown potential in restoring mitochondrial homeostasis and reducing oxidative stress.

## Figures and Tables

**Table 1:** Timeline of key concepts linking Empedocles’ philosophy to the Endosymbiotic theory.

Historical Period/Date	Key Figure(s)	Main Concept/Contribution	Relevance to Endosymbiosis
~495–435 BC	Empedocles of Acragas	Four elements (‘rhizomata’), forces of attraction (Philotis) and repulsion (Neikos), aggregation of parts with differential survival	First conceptual link between merging of distinct units and evolution of new forms
4th century BC	Aristotle	Criticism of Empedocles’ views; emphasized the soul as driver of growth and organization	Highlighted philosophical debate on life origins
1859	Charles Darwin	Natural selection and common descent	Parallel to Empedocles’ concept of survival of certain combinations
1880s–1920s	Anton de Bary, Konstantin Mereschkowsky, Boris Kozo-Polyansky	Early symbiosis theories	Paved the way for endosymbiotic hypothesis
1967	Lynn Margulis	Formalized the endosymbiotic theory for mitochondria and plastids	Established modern scientific basis for mitochondrial origin

## Data Availability

No new data were created or analyzed in this study. Data sharing is not applicable to this article.
